# Enhancing Dementia Care in Rural Communities: A Tele‐rehabilitative Approach through Community‐Centered Technology Integration

**DOI:** 10.1002/alz.087932

**Published:** 2025-01-09

**Authors:** Marguerite Corvini, Jing Wang, Sajay Arthanat

**Affiliations:** ^1^ University of New Hampshire, Durham, NH USA; ^2^ 4 Library Way, Durham, NH USA

## Abstract

**Background:**

Resource‐constrained rural areas face significant challenges in providing access to healthcare resources, especially for older adults, including those living with Alzheimer’s disease and related dementia (ADRD). We seek to address these gaps by equipping six rural community sites in New Hampshire and Maine with tele‐rehabilitative equipment. Libraries and community centers that serves youth and older adults, vital in rural communities, are identified as key partners to advance digital health literacy, equity, and telemedicine services for older adults including those living with ADRD, with the University of [blind for review] Center for Digital Health Innovation (CDHI).

**Method:**

The project includes one hub site, one hub/end‐user site, and four end‐user sites spread across rural communities in New Hampshire and Maine. Each location will receive advanced tele‐rehabilitative tools, such as video conferencing and virtual reality (VR) technologies. The [blind for review] CDHI is responsible for equipment deployment, local staff training, and the formulation of telehealth programs tailored to meet the needs of older adults, particularly those with ADRD. We will obtain feedback from users and staff through focus groups regarding the barriers and facilitators to accessing and integrating the telehealth resources leveraging the strategic role of libraries and community centers in these communities.

**Result:**

The implementation of the project is set to bolster tele‐rehabilitative service capacity across the consortium, potentially impacting around 70,000 rural residents. Expected outcomes include insights into the barriers and facilitators impacting access to specialized healthcare services for older adults with dementia and specific community engagement strategies in rural settings.

**Conclusion:**

This initiative represents a focused strategy to address healthcare and educational disparities experienced by older adults, including those with ADRD, in rural settings. By integrating advanced technology with community engagement, it not only aims to improve the quality of life for these individuals but also sets a precedent for similar rural development programs, striving to close the gap in healthcare and education in resource‐limited areas.

